# Effect of a four-week isocaloric ketogenic diet on physical performance at very high-altitude: a pilot study

**DOI:** 10.1186/s13102-023-00649-9

**Published:** 2023-03-20

**Authors:** Nicolas Chiarello, Bertrand Leger, Mathieu De Riedmatten, Michel F. Rossier, Philippe Vuistiner, Michael Duc, Arnaud Rapillard, Lara Allet

**Affiliations:** 1 Department of Medicine, University Hospitals of Geneva, University of Geneva, Geneva, Switzerland; 2 Clinique romande de réadaptation, Sion, Switzerland; 3 Emergency Department, Hospital Valais/Wallis, Valais, Switzerland; 4 Service of Clinical Chemistry and Toxicology, Central Institute of the Hospitals – HVS, Sion, Switzerland; 5 Department of Medicine, Faculty of Medicine, University of Geneva, Geneva, Switzerland; 6 Institute for Research in Rehabilitation, Clinique romande de réadaptation, Sion, Switzerland; 7 Swiss Olympic Medical Center, Clinique romande de réadaptation, Sion, Switzerland; 8 Valais-Wallis School of Health Sciences, HES-SO, University of Applied Sciences and Arts Western Switzerland, Valais, Switzerland

**Keywords:** Ketogenic diet, Low-carbohydrate, High-altitude, Hypoxia, VO_2_max, Endurance exercise

## Abstract

**Background:**

A ketogenic diet (KD) reduces daily carbohydrates (CHOs) ingestion by replacing most calories with fat. KD is of increasing interest among athletes because it may increase their maximal oxygen uptake (VO_2_max), the principal performance limitation at high-altitudes (1500–3500 m). We examined the tolerance of a 4-week isocaloric KD (ICKD) under simulated hypoxia and the possibility of evaluating ICKD performance benefits with a maximal graded exercise bike test under hypoxia and collected data on the effect of the diet on performance markers and arterial blood gases.

**Methods:**

In a randomised single-blind cross-over model, 6 recreational mountaineers (age 24–44 years) completed a 4-week ICKD followed or preceded by a 4-week usual mixed Western-style diet (UD). Performance parameters (VO_2_max, lactate threshold [LT], peak power [P_peak_]) and arterial blood gases (PaO_2_, PaCO_2_, pH, HCO_3_^−^) were measured at baseline under two conditions (normoxia and hypoxia) as well as after a 4-week UD and 4-week ICKD under the hypoxic condition.

**Results:**

We analysed data for all 6 participants (BMI 19.9–24.6 kg m^−2^). Mean VO_2_max in the normoxic condition was 44.6 ml kg^−1^ min^−1^. Hypoxia led to decreased performance in all participants. With the ICKD diet, median values for PaO_2_ decreased by − 14.5% and VO_2_max by + 7.3% and P_peak_ by + 4.7%.

**Conclusion:**

All participants except one could complete the ICKD. VO_2_max improved with the ICKD under the hypoxia condition. Therefore, an ICKD is an interesting alternative to CHOs dependency for endurance performance at high-altitudes, including high-altitude training and high-altitude races. Nevertheless, decreased PaO_2_ with ICKD remains a significant limitation in very-high to extreme altitudes (> 3500 m).

*Trial registration* Clinical trial registration Nr. NCT05603689 (Clinicaltrials.gov). Ethics approval CER-VD, trial Nr. *2020-00427, registered 18.08*.2020—prospectively registered.

## Introduction

High-fat/low-carbohydrate diets or ketogenic diets (KDs) are an innovative strategy to enhance endurance performance if exercise duration is long enough (e.g., > 4 h) and exercise intensity is low enough (50–60% maximal oxygen uptake [VO_2_max]) [1]. This strategy restricts daily carbohydrates (CHOs) consumption while maintaining low to moderate protein content, thus replacing most calories with fat. There is no standard definition of KD to achieve ketosis because of interindividual variability. CHOs consumption < 30–50 g d^−1^ represents an accurate assessment [2, 3].

From a human evolutionary perspective, fat played a dominant role in energy supply [4]. However, the advent of agriculture shifted the major calorie contributor from fat to CHOs [5].

A century ago, modern science tried to determine the ideal human diet to optimize performance, with several historical studies conducted between 1939 and 1967 [6–8]. Performance was first enhanced in 1939 by giving additional CHOs to individuals with low blood sugar [6]. The association of glycogen depletion with the development of fatigue and glycogen resynthesis with a CHOs-rich diet were later shown by the needle biopsy technique [7]. The idea that only a high-CHOs diet could optimize performance gained credence, with a major effort for optimising glycogen storage. Over time, this led to the most commonly accepted dietary recommendation among athletes: high-CHOs, moderate-protein and low-fat diet [9].

However, in the early 1980s, a link between high-fat diets and exercise capacity was demonstrated. Phinney et al. [10] showed un-impaired performance in patients with a KD [10]. Several studies challenged the approach of glycogen storage optimisation for enhancing endurance performance. There are some indications that high-CHOs consumption may limit athlete’s performance when competing for an extended time. CHOs stores in muscle tissue (300 g), liver tissue (90 g) and the blood stream (30 g) are sufficient for only 1–3 h of activity for endurance athletes [11]. Peak fat oxidation rate occurs in submaximal exercise intensity between 47 and 64% of VO_2_max [1, 12]. Also, well above this CHOs threshold (> 80% VO_2_max), athlete’s performed equally well while eating a high-fat or high-CHOs diet [13]. Empiric observations also indicate well-being with a traditional Inuit diet [14] almost exclusively based on fat and proteins. Furthermore, a growing number of keto-adapted ultra-runner athletes are competing at high levels.

In this context, a new paradigm emerges with the idea to use the virtually unlimited fat store for endurance exercises [1, 5, 15]. The body can adapt to use fat as its primary fuel during submaximal exercise [12] with metabolic adaptation similar to prolonged fasting [4] and increasing the fat oxidation rate (from 0.4–0.6 to 1.2–1.3 g min^−1^) [16]. This probably functions by affecting the mitochondrial respiratory chain [2, 16–19]. Furthermore, a KD leads to a biological ketosis by forcing the liver to produce ketone bodies (KBs) by diverting acetyl-CoA [2]. KBs may positively affect slow-muscle fibers (type I) and negatively affect fast-muscle fibers (type II), which can potentially also enhance endurance exercises [2, 18]. KBs are also suggested to be more energy-efficient than glucose [15, 20].

The concept of positive effects of keto-adaptation on endurance performance is still strongly challenged. Burke et al. [21] investigated the effect of a high-fat diet during a 3-week intensified training. In this study, increased rate of fat oxidation resulted in increased oxygen demand for a given work load, impairing exercise economy [21]. Still today, exercise and sport nutrition guidelines recommend that endurance athletes eat more CHOs (7–10 g kg^−1^ d^−1^) than routine CHOs intake (5–7 g kg^−1^ d^−1^) to optimise muscle glycogen stores [22–24].

High-altitude mountaineering is said to have been invented in the middle of the eighteenth century by H.B. de Saussure. Originally limited to scientists and conquistadors, mountaineering as a sport that really emerged much later. The first Mount Everest ascent without oxygen in 1978 contributed to this aspect. Since then, a plethora of studies have explored the major performance limitations at high altitude. Indeed, diminished inspiratory oxygen pressure (PIO_2_) at high altitude [25] is critical for the delivery of oxygen to tissue. The VO_2_max performance parameter indicates the maximal oxidative metabolic capacity or oxygen supply integrating every step of transport and metabolic capacity of the body [26]. Reduced oxygen delivery at high altitude is responsible for VO_2_max limitation [27].

A KD influences VO_2_max by shifting mitochondrial metabolism capacity. Increasing fat rate oxidation requires greater oxygen consumption, thus leading to higher maximal oxygen supply for maintaining a given exercise load [2]. Some evidence suggests a positive effect of a KD on VO_2_max [21, 28–31] but is contrasted by recent work of Burke et al. [21]. At present, these mixed findings are believed to be due to heterogeneity across studies and/or variability among athletes [32]. Nevertheless, this aspect was never investigated under hypoxic conditions. Despite the mixed effect on VO_2_max, a KD could be a potential performance enhancer in hypoxia. In fact, optimising the fat oxidation rate could give access to the virtually endless fat store and reduce dependence on glycogen. This aspect is particularly important in long hypoxia training such as mountaineering. Furthermore, hypoxia is known to induce a reduction in CHOs oxidation when CHOs are ingested before exercise, thus reinforcing the use of fat at high altitudes [33, 34]. This observation is supported by the subjective benefit of a high-fat diet in high altitude praised by the extreme high-altitude mountaineer Erhard Loretan (e.g., at Everest base camp in 1986).

According to this information, we hypothesized that a 4-week KD would have positive effects on VO_2_max in healthy, recreational mountaineers during a maximal graded performance test under simulated hypoxic conditions. Various types of KD are described [3]. In our study, we focused on the isocaloric KD (ICKD) in which calories are in line with total energy expenditure.

## Methods

This pilot study was a single, blinded, randomised cross-over clinical trial. The study took place in Switzerland at the Clinique romande de réadaptation (Sion). The protocol was approved by CER-VD (Project-ID: 2020-00427).

### Study aim

This pilot study aimed to (1) assess the tolerance of a non-standardized 4-week ICKD in healthy, recreational mountaineers, (2) assess the possibility of evaluating participants’ ICKD performance benefits under hypoxic conditions by a maximal graded exercise bike test and (3) gather data regarding the benefit of a 4-week ICKD on VO_2_max during a maximal graded performance test under simulated hypoxic conditions. Furthermore, data concerning lactate threshold (LT) values (P_LT_, HR_LT_), peak values (P_peak_, HR_peak_), subjective Borg rating of perceived exertion (RPE) and oxygenation status (blood gases) were recorded.

### Participants

Eight recreational mountaineers were recruited to participate. Inclusion criteria were familiarity with altitude (> 2500 m above sea level) and males/females 20–45 years old. Exclusion criteria were high training load (such as professional athletes) or new planned training and dietary restrictions. Participants were enrolled after giving their signed informed consent. Mountaineering level was reported as beginner for participants with no to little experience in mountaineering, medium for those who regularly experienced high altitudes, and experts who regularly mountaineered.

We did not calculate a sample size for this study because this was a pilot study to gather information about diet tolerance/acceptance, the feasibility of the testing procedure and preliminary data on the benefit of an ICKD diet for VO_2_max in healthy persons.

### Study procedure

Individuals who gave signed informed consent were invited for a consultation during which a medical doctor explained the standardisation of the performance tests and the dietary protocol. In addition, the participant’s ability to perform a maximal graded performance test was assessed with the physical activity aptitude questionnaire (Q-AAP) [35]. We assessed participants’ ability for a ICKD and exposure to hypoxia by two other self-developed questionnaires using known contra-indication to KD [36], previously experienced exposure to hypoxia and predisposing factors to acute mountain sickness [37].

The participant was then asked to perform a maximal graded exercise bike test (performance test) to assess their baseline performance under normoxic conditions (T0N) and, 4 weeks later, under hypoxic conditions (T0H). This test was conducted under supervision by a sport scientist and a medical doctor. The medical doctor was also responsible for collecting an arterial blood sample after a 5-min rest post-exercise.

After this test, participants were randomly assigned to group A or group B with a block size of 4 by a collaborator who was not involved in the study protocol. The RALLOC function of Stata was used. Each participant received a with the group attribution, which allowed for blind the investigators. Group A began with a 4-week UD (T1) followed by a 4-week ICKD (T2), and group B began with a 4-week ICKD (T1) followed by a 4-week UD (T2).

**Fig. 1:**
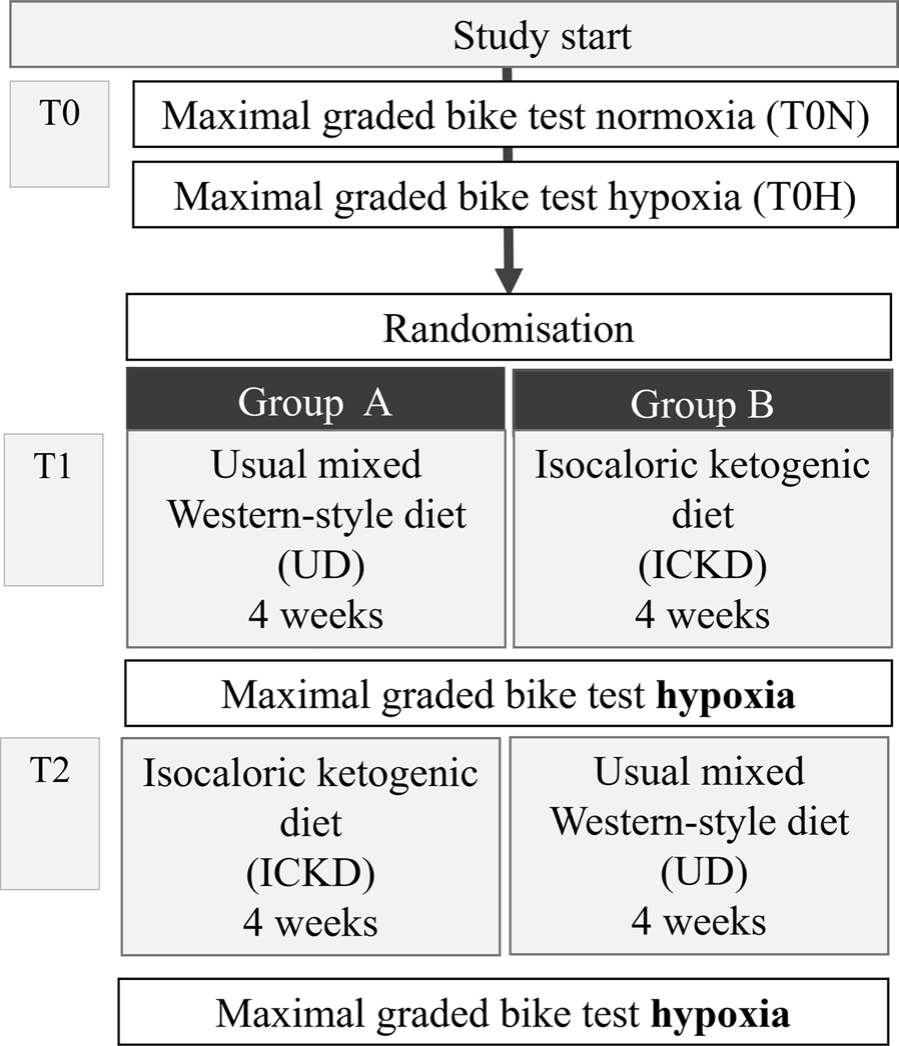
Study design

Each 4-week diet period was terminated with a performance test under hypoxic conditions (same procedure as at baseline but under hypoxic conditions). We used a normobaric (940–980 hPa) hypoxic (FIO_2_ = 12.7–12.9%) room simulating an altitude of ~ 4500 m. The study design is represented in Fig. 1.

### Maximal graded exercise bike test

The initial resistance was 60–90 W depending on training status and sex. The resistance was increased every 3 min by 30 W until exhaustion. Interruption conditions were a clear decrease in cycling frequency (< 70 min^−1^) or a complete stop of cycling [38]. Oxygen respiratory flow (VO_2_), carbon dioxide respiratory flow (VCO_2_), heart rate (HR) and delivered power (P) were measured (Metalyzer 3B, Cortex) until the test’s interruption. Maximal values are defined as peak values. In addition, capillary blood lactate (B-Lac) level was measured 30 s before the end of each increase in resistance. Finally, we used Borg RPE [39] at the end of the test to assess participant subjective perceived exertion of the physical work.

Hypoxia during the performance test was achieved in a simulated altitude facility (hypoxic room) with a hypoxic generator (ATS altitude, Sydney, Australia) by lowering the fraction of inspired oxygen (FIO_2_), which simulates a very-high-altitude of 4500 m.

### Study intervention

#### Four-week ICKD

The ICKD definition was based on the works of Sansone et al. [2] and Trimboli et al. [3]. We defined ICKD as a daily CHOs ingestion < 30–50 g d^−1^, without any limitation in fat consumption. Participants self-selected their own diet based on a list of advised and forbidden foods developed to fit the definition of ICKD. There were no instructions to limit calories. Regular contact was maintained with all participants during the study with every time available to answer participants’ dietary questions. The maximal graded bike test was planned after an adaptation period of 27 days.

#### Four-week UD

There were no limitations on food consumption. Participants were instructed to eat as close as possible to their usual diet.

Participants were instructed to track their 4-week ICKD and UD by using the analysis program MyFoodRepo© (EPFL, Switzerland), a user-friendly smartphone application. The database used by the application is based on Switzerland’s foods and is in constant development. Food intake was manually reported on a daily basis by an investigator for diet monitoring. Participant adherence to ICKD was checked by their daily CHOs intake. If daily CHOs intake was > 50 g and if the β-hydroxybutyrate (β-OHB) level was < 170 μmol L^−1^, data for the participant were excluded from the statistical data analysis and the study.

### Blood analysis

An arterial blood sample was taken after a 5-min rest at the end of each performance test. Under hypoxic conditions, the participants remained in the hypoxic room for blood collection. The blood samples were analysed on an ABL800 FLEX blood gas analyzer (Radiometer, Denmark) within 10 min for partial arterial oxygen pressure (PaO_2_ [kPa]), partial arterial carbon dioxide pressure (PaCO_2_ [kPa], pH, and bicarbonate concentration (HCO_3_^−^ [mmol L^−1^]) automatically calculated by using the Henderson–Hasselbalch equation.

Venous blood was sampled after each test. Venous blood was placed into a perchloric acid-tube and frozen at − 80 °C. All samples were analyzed within 3 months for β-OHB and acetoacetate (AcAc) with enzymatic analysis [40]. Reference values (percentile 2.5–97.5) provided by the laboratory (Lausanne university hospital, Switzerland) were for β-OHB, 58–170 μmol L^−1^, and AcAc, 18–78 μmol L^−1^.

### Statistical analysis

We used descriptive statistical analysis for (1) diet tolerance, (2) performance values under normoxic and hypoxic conditions and (3) performance values under a UD and ICKD. Performance test results under normoxic and hypoxic conditions are expressed as mean (SD). Because of limited sample size (n = 6), no confidence intervals or p-values were calculated.

To assess diet tolerance, we analysed the number of dropouts and reported side effects. We assessed diet tolerance by calculating median daily values for CHOs, fat and protein content and energy expenditure for all participants, then calculated median daily CHOs, fat, and protein consumption and energy expenditure for all participants. Further blood analysis of KBs including β-OHB and AcAc were assessed for diet tolerance.

Feasibility of maximal graded exercise was assessed by comparing the values under normoxia and hypoxia. These values were then expressed as percentage difference in “median” (“minimal values” to “maximal value”). We then checked whether these values agreed with previously reported performance decreases under acute hypoxia [41–43].

The effect of ICKD on performance is also expressed as percentage differences in median (range), calculated by comparing the values of performance parameters after the ICKD diet and the UD diet. For Group A, UD values are T1. For Group B, UD values are T0H. VO_2_max performance parameters after ICKD (hypoxia) compared to UD (hypoxia) were the primary outcome. As a secondary outcome, performance parameters such as LT values (P_LT_, HR_LT_), peak values (P_peak_, HR_peak_), subjective values (Borg RPE) and oxygenation status (blood gas values) were analysed. LT determination was based on the D_max_ model established in 1992 [44], which uses the maximal perpendicular distance from the line connecting the start with the endpoint of the lactate curve. We used the “modified D_max_ threshold” (D_mod_), which is an updated D_max_ model by Bishop et al. [45]. This model eliminates the effect of start intensity and maximal effort and determines the LT as the moment when a rapid change in the inclination of the blood lactate curve occurs. This situation matches the maximal lactate steady state reflecting the anaerobic threshold [46].

## Results

### Diet tolerance

**Fig. 2:**
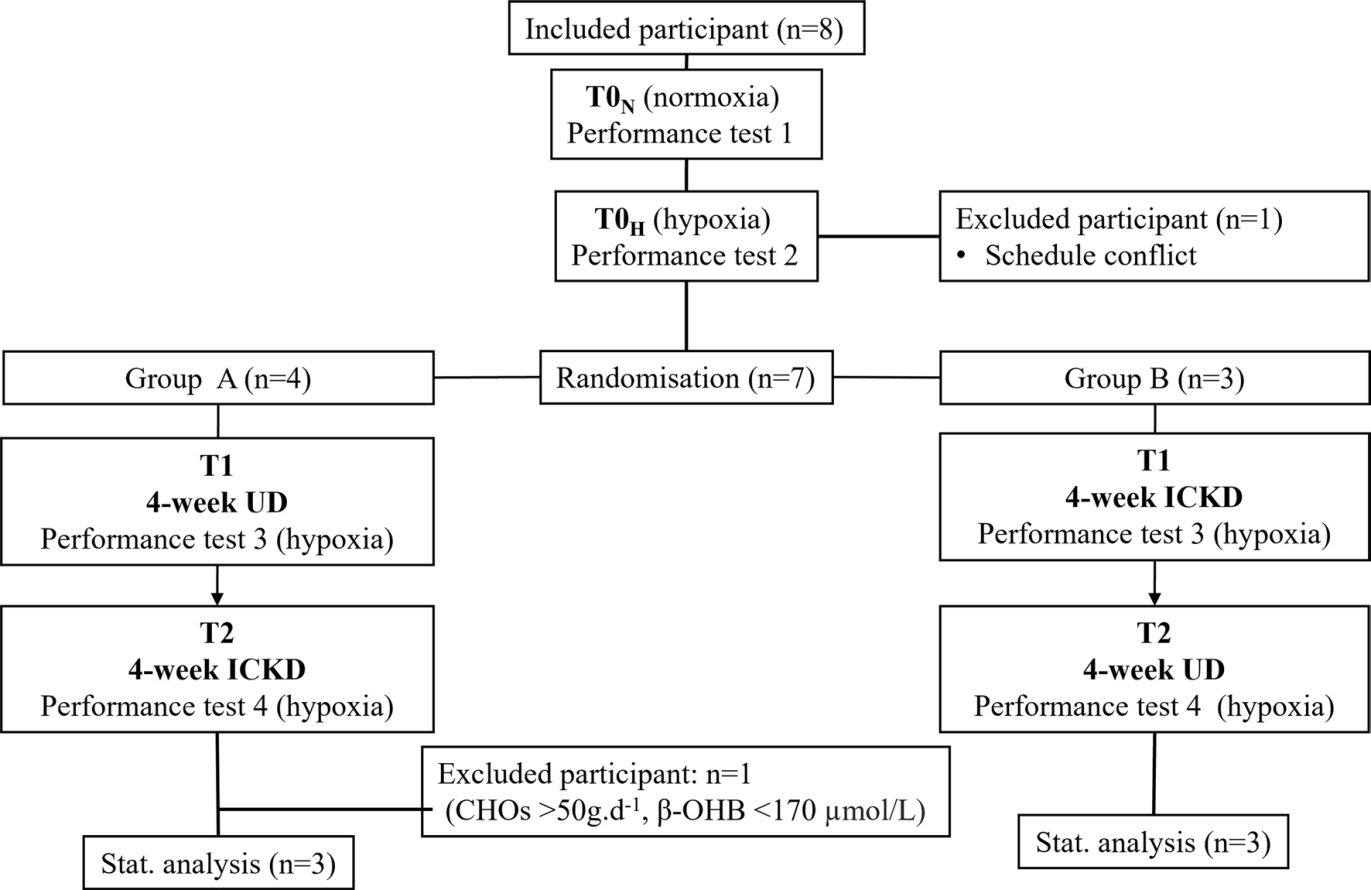
Flow of participants in the study. ICKD: isocaloric ketogenic diet; UD: usual mixed Western-style diet; CHOs: carbohydrates; β-OHB: β-hydroxybutyrate level

At the beginning, 8 participants were enrolled (4 males). One participant dropped out after the second performance test because of a schedule mismatch rather than a regimen intolerance. In addition, the data of another participant were excluded from data analysis because of abnormally high daily CHOs intake (> 50 g d^−1^) and low β-OHB level (< 170 µmol/L), which led to suspecting invalid data (Fig. 2).

Participants were aged 24–44 years (median 29 years) and the median BMI was 22.8 kg m^−2^.

Frequently reported side effects were weight loss and gastrointestinal disorders. After ICKD, subjective exercise load perception by the RPE scale [39] were similar to reference values for two participants, increased for three participants and decreased for one participant. Nutrition features with the UD and ICKD are presented in Table 1.

**Table 1:** Nutrition features of participants with a usual mixed Western-style diet (UD) and isocaloric ketogenic diet (ICKD)

	UD			ICKD		
	Median	Min	Max	Median	Min	Max
CHOs (g d^−1^)	197	152	321	40	21	49
Protein (g d^−1^)	82	51	122	97	51	169
Fat (g d^−1^)	93	63	140	143	43	164
Energy (Kj d^−1^)	7774	6366	12,603	8496	4475	9841
β-OHB (μmol/L)	44.0	41.0	65.0	288.0	190.0	462.0
AcAc (μmol/L)	22.5	6.7	33.0	80.0	59.0	132.0

Recorded data for UD and ICKD diet characteristics with median (range) showing adherence to the ICKD diet. Reference values (percentile 2.5–97.5) provided by the laboratory (CHUV, Switzerland) are for β-OHB, 58–170 μmol L^−1^, and for AcAc, 18–78 μmol L^−1^. *CHOs* carbohydrates, *β-OHB* β-hydroxybutyrate, *AcAc* acetoacetate

#### UD and ICKD follow-up

The 4-week UD diet was characterized by a median CHOs intake of 197 g d^−1^. The median post-UD β-OHB level 44.0 μmol/L and AcAc level 22.5 μmol/L were within reference values. With the 4-week ICKD, the median CHOs intake was 40 g d^−1^. The median post-4-week ICKD β-OHB value 288.0 μmol/L and AcAc value 80.0 μmol/L were above reference values fixed by the laboratory.

One participant showed excess CHOs daily intake and too low β-OHB blood level. His median post-4-week UD CHOs intake was 259 g d^−1^ and 4-week ICKD intake 67 g d^−1^. The β-OHB/AcAc blood content was 41.0/23.0 and 152.0/56.0 mmol L^−1^. This participant was not included in the statistical analysis.

### Feasibility of the maximal graded exercise bike test under hypoxia

We used expected and previously characterised performance decreases under hypoxia [41–43] to evaluate the feasibility of the performance test and trustworthiness of the recorded values. We found no adverse events related to the combination of a 4-week ICKD, hypoxia exposure and maximal graded exercise bike test.

#### Effect of hypoxia

The results of the performance test under normoxic conditions at baseline are presented in Table 2. The measured effect of hypoxia is expressed as the median difference with normoxic values in percentages. Median performance values decreased for VO_2_max (− 27.1%), P_LT_ (− 28.6%) and P_peak_ (− 22.4%) under hypoxic conditions. Arterial blood samples showed a reduction in median PaO_2_ by − 50.9%. Median values for pH, PaCO2 and HCO3- all remained unaffected.

**Table 2:** Effect of hypoxia alone on performance values

	Normoxia	Effect of hypoxia (%)			
	Mean (SD)	Median	Min		Max
VO_2_max (ml kg^−1^ min^−1^)	44.6 (5.8)	− 27.1	− 36.5	− 20.3	
P_LT_ (W)	203 (64)	− 28.6	− 34.6	− 17.9	
P_peak_ (W)	266 (65)	− 22.4	− 30.8	− 12.8	
HR_LT_ (beat min^−1^)	156 (23)	2.0	− 7.1	17.6	
HR_peak_ (beat min^−1^)	183 (17)	− 5.4	− 15.0	− 1.9	
B-Lac_peak_ (mmol L^−1^)	10.7 (2.2)	− 0.8	− 28	16.1	
PaO_2_ (kPa)	17.8 (2.2)	− 50.9	− 71.1	− 12.2	
pH	7.24 (0.06)	0.15	− 0.59	2.22	
PaCO_2_ (kPa)	3.8 (0.2)	− 9.6	− 19.5	5.9	
HCO_3_^−^ (mmol L^−1^)	11.9 (2.1)	0.0	− 0.2	0.1	

Maximal graded exercise test values under normoxic conditions for baseline were compared with performance values under hypoxic conditions

Normoxia baseline values (T0N) are compared with hypoxia values (T0H) assessing the effect of hypoxia. *VO*_*2*_*max* maximal oxygen uptake, *LT* lactate threshold, *P* power, *HR* heart rate, *B-Lac* blood lactate, *PaO*_*2*_ partial oxygen arterial pressure, *PaCO*_*2*_ partial carbon dioxide arterial pressure

### ICKD effect on performance

Performance test results under hypoxic conditions at baseline are in Table 3. Keeping the cross-over design, hypoxic baseline values (UD) are at T1 for Group A and T0H for group B. The effect of ICKD on performance test values was assessed by comparing parameter values at T1 with T2 (Group A) and T0H with T1 (Group B). A return to the normal situation could be assessed only in group B by comparing the performance values of T0H and T2.

**Table 3:** Effect of ICKD under hypoxic conditions on performance values

	Hypoxia	Effect of ICKD (%)			
	Mean (SD)	Median	Min		Max
VO_2_max (ml kg^−1^ min^−1^)	31.6 (3.4)	7.3	− 16.8	25.5	
P_LT_ (W)	147 (47)	0.7	− 21.5	46.6	
P_peak_ (W)	205 (48)	4.7	− 7.1	11.1	
HR_LT_ (beat min^−1^)	160 (22)	7.5	− 5.4	23.8	
HR_peak_ (beat min^−1^)	171 (15)	3.6	− 2.6	15.2	
B-Lac_peak_ (mmol L^−1^)	10.3 (3.0)	− 8.8	− 40.9	17.1	
PaO_2_ (kPa)	9.0 (3.4)	− 14.5	− 32.1	− 11.5	
pH	7.28 (0.96)	0.28	0.08	1.62	
PaCO_2_ (kPa)	3.5 (0.4)	2.1	− 23.3	11.8	
HCO_3_^−^ (mmol L^−1^)	12.0 (3.1)	− 0.1	− 0.3	0.1	

Hypoxic (T0H) performance values were used for baseline and were compared with performance values from a maximal graded exercise test performed after a 4-week ICKD under hypoxic conditions

*ICKD* isocaloric ketogenic diet, *UD* usual mixed Western diet, *VO*_*2*_*max* maximal oxygen uptake, *LT* lactate threshold, *P* power, *HR* heart rate, *B-Lac* blood lactate, *PaO*_*2*_ partial oxygen arterial pressure, *PaCO*_*2*_ partial carbon dioxide arterial pressure

#### Effect of ICKD

Median performance values increased for VO_2_max (+ 7.3%), P_peak_ (4.7%), HR_peak_ (+ 3.6%) and P_LT_ (+ 0.7%) but with large interindividual variability (Table 3). Median bLa_base_ and bLa_peak_ values decreased by − 21.0% and − 8.8%. Median PaO_2_ decreased − 14.5%. Values for other parameters, pH, PCO_2_ and HCO_3_, could be considered stable.

In Group B, the cross-over design allowed for assessing a return to the normal situation when switching again from a 4-week ICKD to a 4-week UD. So we compared T0H and T2 values for the three participants in group B, which should theoretically be the same values. We expressed results as a percentage difference between T0H and T2. Of note, only median PaO2 (+ 1.4%) returned to the initial value. We did not observe a return to initial values for median VO_2_max (+ 18.1%), Ppeak (− 8.3%), or PLT (+ 3.3%).

#### Individual values

**Fig. 3:**
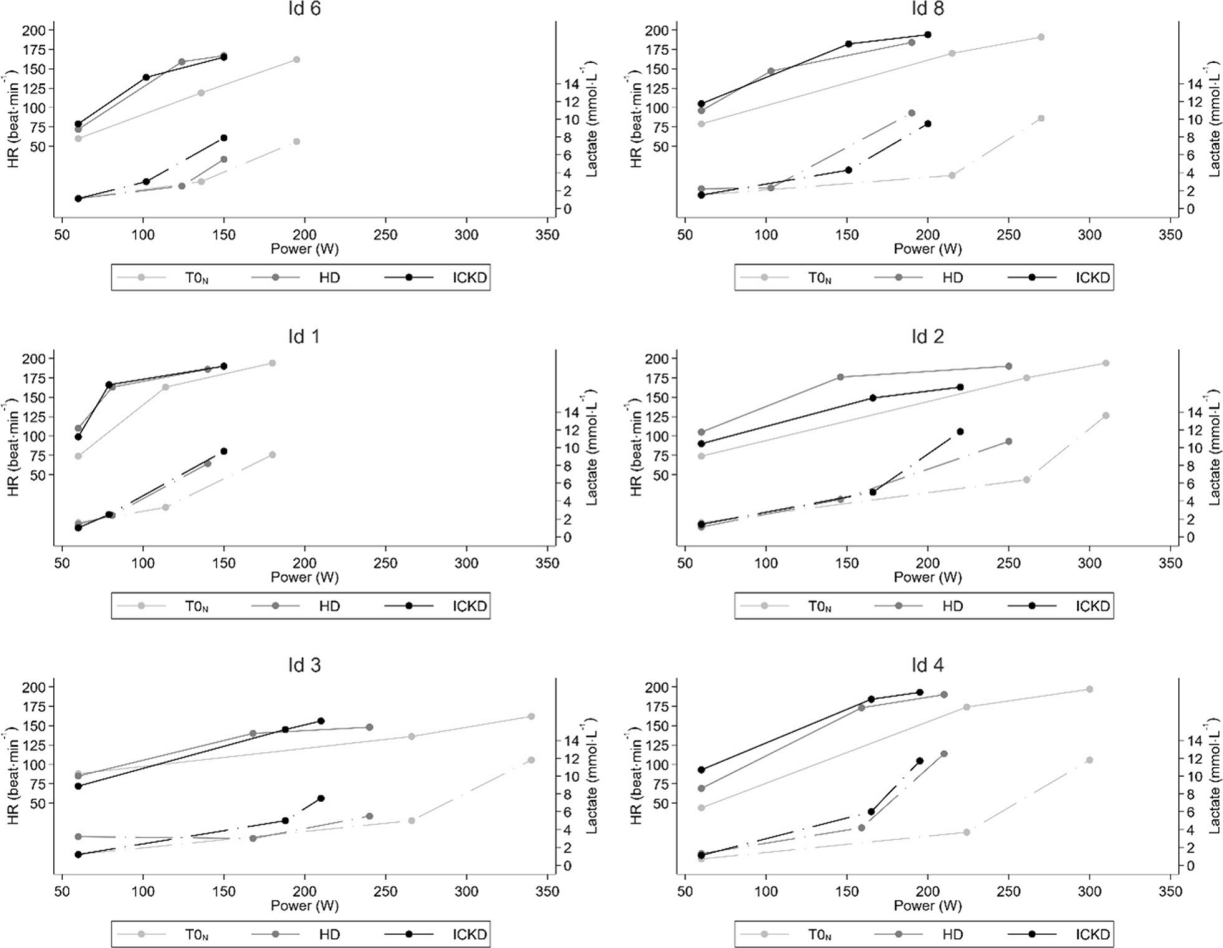
Effect of ICKD on performance test. ICKD B-Lac and HR curves were drawn for T0N, UD, and ICKD. The first point represents the values at the beginning of the test. The second point is the value at lactate threshold and the third point is the maximal or peak value. ID: participant number

Individual performance and blood gas parameters at baseline (T0N) after UD and after ICKD are in Table 4. The 6 participants could be separated as showing a positive effect of ICKD or not (Table 5). Four participants showed an increase in VO_2_max and P_peak_. P_LT_ was used for further dividing participants. Figure 3 shows B-Lac and HR curves after UD or ICKD, as well as at T0N for every participant. Nr. 6 (Id 6) and Nr. 8 (Id 8) showed improvement in endurance and peak performance parameters. Nr. 1 (Id 1) and Nr. 2 (Id 2) showed improvement in peak performance parameter. Nr. 3 (Id 3) and Nr. 4 (Id 4) showed little to no response or a clear worsening of the performance parameter**.** One participant (no. 2) showed a particularly large decrease in HR_peak_ (− 14.9%) with hypoxia exposure. ICKD clearly allowed for a return to normality for HR_peak_ values (+ 13.1%) as also confirmed by the cross-over analysis (− 14.2%).

**Table 4:** Values for individual participants comparing performance after a UD and after an ICKD

	Test	Nr. 1	Nr. 2	Nr. 3	Nr. 4	Nr. 6	Nr. 8
Sex		F	M	M	M	F	F
Age (years)		29	27	44	24	29	28
BMI (kg m^−2^)		22.3	23.2	24.6	23.4	21.3	19.9
Mountaineering level		Medium	Medium	Medium	Expert	Medium	Expert
VO_2_max (ml kg^−1^ min^−1^)	T0N	36.8	48.8	46.2	50.7	38.0	47.2
	UD	26.6	31.0	29.5	42.8	28.5	32.3
	ICKD	28.8	38.9	24.5	38.4	33.6	34.4
P_LT_ (W)	T0N	114	261	266	224	136	215
	UD	81	186	182	159	89	103
	ICKD	79	146	168	165	124	151
P_peak_ (W)	T0N	180	310	340	300	195	270
	UD	140	240	194	210	123	190
	ICKD	150	250	186	195	140	200
HR_LT_ (beat min^−1^)	T0N	163	175	136	174	119	170
	UD	163	186	129	173	140	147
	ICKD	166	176	140	184	159	182
HR_peak_ (beat min^−1^)	T0N	194	194	162	197	162	191
	UD	186	165	152	190	159	184
	ICKD	190	190	148	193	167	194
bLa_peak_ (mmol L^−1^)	T0N	9.2	13.6	11.8	11.8	7.5	10.1
	UD	8.2	13.1	9.3	12.5	5.4	10.7
	ICKD	9.6	10.7	5.5	11.7	5.5	9.5
RPE	T0N	10	17	17	17	11	17
	UD	11	20	15	16	13	18
	ICKD	15	20	15	17	15	16
PaO_2_ (kPa)	T0N	18	13.6	19	25.5	14	16.9
	UD	15.8	7.0	7.7	11.7	8.1	8.4
	ICKD	7.7	6.0	6.8	7.9	6.0	7.2
pH	T0N	7.22	7.14	7.26	7.26	7.34	7.24
	UD	7.21	7.21	7.42	7.20	7.38	7.28
	ICKD	7.25	7.33	7.42	7.22	7.45	7.29
PaCO_2_ (kPa)	T0N	3.6	3.9	3.7	3.5	4.0	4.0
	UD	2.9	3.4	3.2	3.7	3.8	3.7
	ICKD	3.0	3.8	3.5	2.9	3.9	3.1
HCO_3_^−^ (mmol L^−1^)	T0N	10.8	9.5	12	11.3	15.6	12.3
	UD	8.4	10	15	10.5	16.5	12.4
	ICKD	9.4	14.7	16.8	8.5	20.2	10.9

Only VO_2_max, P_LT_ and P_peak_ values are shown for baseline T0N

*T0N* baseline test under normoxia, *UD* values after usual mixed Western-style diet under hypoxia, *ICKD* isocaloric ketogenic diet under hypoxia, *VO*_*2*_*max* maximal oxygen uptake, *LT* lactate threshold, *P* power, *HR* heart rate, *bLa* blood lactate, *RPE* Borg rating of perceived exertion, *PaO*_*2*_ partial oxygen arterial pressure, *PaCO*_*2*_ partial carbon dioxide arterial pressure, *Nr*. subject number

**Table 5:** Effect of ICKD on performance parameters

Type	Nr	VO_2_max	P_LT_ and HR_LT_	P_peak_	Remark
Positive effect of ICKD	6;8	↑	↑	↑	ICKD conferred improvement regarding endurance and peak performance parameters
	1;2	↑	↓	↑	ICKD had positive effects despite a decrease in LT parameter values. Indeed, P_peak_ and VO_2_max confirmed a benefit
Negative effect of ICKD	4	↓	↑	↓	ICKD conferred little to no response. UD and ICKD values for performance tests can be considered equal
Non-assessable	3	↓	↓	↓	Clear worsening of test performance

*ICKD* isocaloric ketogenic diet, *UD* usual mixed Western-style diet, *VO*_*2*_*max* maximal oxygen uptake (ml min^−1^ kg^−1^), *P*_*LT*_ power at lactate threshold (W), *P*_peak_ peak power (W), *HR*_*LT*_ heart rate at lactate threshold (beat min^−1^), *Nr.* subject number

## Discussion

This novel study assessed the tolerance of an ICKD and the feasibility of a bike performance test under hypoxic conditions and gathered preliminary data on the effect of shifting from a UD to an ICKD on VO_2_max under simulated very-high-altitude conditions. We used a maximal graded exercise bike test to assess endurance parameters and post-exercise arterial blood samples to assess oxygenation and pH status. We hypothesized that reduced CHOs intake would increase VO_2_max performance values under hypoxia. To our knowledge, these aspects were never investigated before. We analysed data for all 6 participants. Mean VO_2_max in the normoxic condition was 44.6 ml kg^−1^ min^−1^. Hypoxia led to decreased performance in all participants. With the ICKD diet, median values for PaO_2_ decreased by − 14.5% and VO_2_max by + 7.3% and P_peak_ by + 4.7%.

### Diet tolerance

All participants except one could complete the ICKD. The recorded CHOs values for this participant were beyond the limitation and therefore the data were excluded from analysis. This situation emphasizes the need for a strict diet follow-up and blood KB analysis. Another participant dropped out because of a personal schedule mismatch, which underlines the importance of high motivation and collaboration between researchers and participants in particular because of the high number of maximal graded exercise bike tests with standardized time between the tests. Overall, only minor adverse events such gastrointestinal complaints at the beginning of the diet or weight loss were reported, but none of the participants had to stop the ICKD.

The median energy intake for UD and ICKD were below the typical energy requirements [47], so participants were in negative energy balance.

Moreover, we identified two major difficulties for participants in following the ICKD. First, high exercise load training was difficult during the first weeks of the ICKD diet. Indeed, we found a subjective performance drop at the beginning of the new diet because of ICKD adaptation [48]. Second, participants also reported a subjective social impact during the ICKD.

### Evaluating the ICKD benefits on performance under hypoxic conditions

We evaluated ICKD-induced performance benefits under hypoxic conditions by using a maximal graded exercise bike test. The observed decrease in median values for the performance parameters VO_2_max, P_LT_, P_peak_ and PaO_2_ when exposed to hypoxia strengthen the reliability of results and the feasibility of our protocol.

#### Effect of hypoxia

For every participant, hypoxia induced a clear decrease in median VO_2_max by 13 ml kg^−1^ min^−1^ (− 27.01%), P_peak_ by 60 W (− 22.4%), and PaO_2_ by 8.8 kPa (− 50.9%) (Table 2). This was expected because the primary limitation for VO_2_max under hypoxic conditions is oxygen tissue availability [49]. Indeed, significant decreases in VO_2_max were previously reported with acute exposure to hypoxia [41–43]. This situation results from decrease in barometric pressure with increasing altitude. Therefore PIO_2_ and, consequently, oxygen transport decrease [41, 50]. A research compilation by Robergs and Robert [51] reported a mean decrease of 8.7% per 1000 m in VO_2_max. Nevertheless, an average value cannot be expressed because of high inter-individual variation in the reduction in VO2max depending on sea level VO_2_max, sex, sea level LT and lean body mass [27]. This observation may explain the large range in VO_2_max decrease (− 36.5 to − 20.3%) in our study. Furthermore, we found no significant difference in arterial blood pH, PaCO2 and HCO3.

### Recorded data

#### Effect of ICKD on maximal graded exercise test

Improved median VO_2_max (+ 7.3%) and P_peak_ (+ 4.7%) with a 4-week ICKD under hypoxic conditions in 4 of 6 participants (Tables 3, 4) do not allow for concluding on the effects of the KD. We found no performance marker systematically affected by ICKD. This recorded improvement was similarly reported in part under normoxia [21, 32]. VO_2_max reflects the cardiorespiratory fitness [52] and is considered a gold standard for measuring aerobic metabolism [53]. A greater VO_2_max indicates greater endurance capacity. Actual known factors affecting VO_2_max are age, sex, genetics, body composition, state of training and mode of exercise [54].

The KD has been newly identified as a potential positive factor for VO_2_max [28, 30, 31] by shifting mitochondrial metabolism [17, 19]. These studies were summarized by Bailey et al. [32]. The authors suggested that several factors such as genetics, trainability and or chronic substrate utilization may be affected by KD, which thus might increase VO_2_max. The positive effect of the ICKD on VO_2_max under hypoxic conditions we observed strengthens the idea that a high-fat diet might be beneficial for endurance exercise under acute hypoxia exposure.

In addition, other performance LT values were improved in two participants considered ICKD responders (Table 5). As shown in Fig. 3, B-Lac kinetics were lower with the ICKD than UD. Points for LT and maximal value shifted to the right (right curve-shifting). HR kinetics were higher with the ICKD than UD. Points for LT and maximal values were at higher power (left curve-shifting). LTs are performance indicators strongly correlated with endurance performance [46]. They represent the aerobic–anaerobic transition because lactate kinetics are highly related to the metabolic rate and less to oxygen availability [46]. A higher workload to a given blood lactate concentration can be interpreted as improved endurance capacity [55]. Previous studies have reported a shift in the B-Lac curve to higher workloads under KD conditions [56]. This phenomenon is still not fully understood but may be due to an association with decreased glycolysis rate or limited lactate efflux from muscle due to reduced blood buffering capacity [28, 56]. Furthermore, depleted glycogen stores (due to an ICKD, for example) is a known factor leading to lower blood lactate concentration at the same work rate [57].

In this context and considering key performance parameters (VO_2_max, P_LT_, HR_LT_ and P_peak_), we separated the 6 participants into two groups: a group showing a positive effect of the ICKD and a group showing no benefits or even negative effects of the ICKD. The group with a positive effect of the ICKD showed a right-shift in B-Lac kinetics (Fig. 3) which can be interpreted as better performance [55]. Peak values (P_peak_ and VO_2_max) also demonstrated better performance. A left-shifting HR curve showed a higher HR work range with the ICKD. For the second group, the intervention had little to no effect, and ICKD and UD tests were considered equal. One participant showed clearly worsened global ICKD performance at every time point, with no clear explanation. Several factors that could explain this performance decrease include a “bad shape day” or the influence of the ICKD on age-related VO_2_max factors such as a decline in maximal heart rate, stroke volume, fat-free mass and arterio-veinous oxygen differences [58].

#### Effect of ICKD on blood gas parameters

With the ICKD, post-exercise PaO_2_ decreased in all participants (median − 14.5%), a response confirmed in the cross-over group B when returning to the UD (+ 14.5%). A physiological approach can explain the ICKD-related hypoxemia. PaO_2_ is determined by alveolar PO_2_ (PAO_2_), ventilation, diffusion capacity of the lung and perfusion by the heart. PAO_2_ depends on the respiratory gas-exchange ratio (RER). RER is the ratio between CO_2_ pulmonary output ($\dot{V}CO_{2}$) and O_2_ uptake ($\dot{V}O_{2}$) expressed as $RER = \frac{{\dot{V}CO_{2} }}{{\dot{V}O_{2} }}$, which can be included in the alveolar air equation $PAO_{2} = PIO_{2} - (\frac{{PaCO_{2} }}{RER})$. RER depends on the steady state (e.g., resting state) for the food metabolized [59]. An ICKD increases fat oxidation and lowers RER (~ 0.7), and consequently PAO_2_ and PaO_2_ decrease [59]. Furthermore, the maximal graded test result is not a steady state, and RER varies with exercise. Within a few minutes into recovery, RER decreases to < 0.7 as ventilation declines and the CO_2_ store re-increases [60]. Decreased ventilation could also affect PaO_2_, as observed in respiratory failure, a well-known process in diabetic ketoacidosis. Ketosis generates a respiratory response in the form of hyperpnea [61], which leads to respiratory muscle fatigue (known as Kussmaul respiration) [62]. An ICKD could lead to a mismatch of the lung maintaining PaO_2_ by a form of respiratory muscle fatigue due to KBs.

Alternative explanations for the effect of heart perfusion on PaO_2_ are not relevant. Although KBs can influence heart flow, they increase rather than decrease the hydraulic efficiency of the heart [18]. The higher heart flow rate leads to an increase in pulmonary venous blood admission. Finally, CHOs are known to increase PaO_2_ at high altitude by increasing the relative production of carbon dioxide and increasing the drive for ventilation [63].

Consistent with a previous study by Hansen et al. [60], ICKD worsened the hypoxemia in our simulated very-high to extreme altitude (> 3500 m). This is a relevant limitation of using the ICKD above a very-high-altitude [50, 64], whereas at high-altitude (1500–3500 m), PaO_2_ is significantly diminished but with only minor impairments in oxygen transport (SaO_2_ > 90%) [50]. Therefore, an ICKD could be used for this altitude.

Arterial blood sampling also showed non-significant effects of the ICKD on pH (+ 0.278%) post-exercise, which is below analytical precision. Increased pH could be expected because KBs are acids known to induce ketoacidosis [65, 66]. Nevertheless, Carr et al. contrasted these earlier beliefs by describing the minimal effects of KD on acid base status in elite athletes [30]. They also reported no statistical blood pH differences between high CHOs versus high fat content pre- and post-exercise. Blood pH stability may be due to increased exercise-induced ventilation rate and so would increase the pH.

In conclusion, our preliminary data showed a benefit of ICKD on performance parameters. We found a positive increase in VO_2_max (primary outcome) and LT performance parameters (secondary outcome). ICKD intervention decreased PaO_2_, which is consistent with previous findings [60] and may limit the use of an ICKD in very-high-altitude sports.

### Further research

Further characterisation of the ICKD benefits at high-altitude (1500–3500 m) is needed. Despite ICKD-induced hypoxemia, this may not impair oxygen delivery at this altitude. Furthermore, diet modifications (CHOs vs. fat) could be an interesting path for improving acclimation or performance at high-altitude and preventing acute mountain sickness. PaO_2_ analysis during the whole effort would be needed for a complete assessment of the effect of an ICKD on blood gas values. The cross-over design of the study is pertinent to assess the effectiveness of an ICKD.

The previous experience of participants with KD-like diets seems to be an effective supplementary inclusion criterion for diet tolerance. Moreover, whether subjective perception of KD tolerance matches performance improvements would be interesting and might help predict whether a person would show a positive effect of ICKD or not. This would be possible with a simple assessment by a questionnaire without a maximal graded performance test.

### Strengths and limitations

Our study assessed for the first time the effect of KD implementation in simulated very-high-altitude performance test. We used a maximal graded exercise bike test with a primary outcome of the effect on VO_2_max. However, with a sample of 6 participants, we focused on individual values, profiling, and trends. The negative energy balance observed without recorded data on body mass at the end of KD limits concluding on its effect. In addition, our design also limits the blood gas kinetics view during the performance test. Moreover, our findings were obtained in acute simulated hypoxia, which limits practical implications for the real-life high-altitude condition.

## Conclusions

The present protocol shows the feasibility of evaluating the benefits of an ICKD on recreational athlete performance by a maximal graded exercise test under hypoxia conditions. Our study successfully combined ICKD, hypoxia and maximal graded exercise, which shows the feasibility of the present protocol. The pilot data showed improved VO_2_max with the ICKD under hypoxia in 4 participants. Nevertheless, this study does not allow to make any final conclusions about the benefit of ICKD. KD remains an interesting alternative to CHOs dependency for endurance performance. This regimen may be interesting for endurance exercises at high-altitude (1500–3500 m) [67] with only minor impairment in arterial oxygen transport [50]. It could concern typical high-altitude exercises such as high-altitude training [67], high-altitude races [68], trail-running, mountaineering and ski-touring [69].

## Data Availability

The data that support the finding of this study are available on request from the corresponding author (N.C.). The data are not publicly available due to legal restriction of the rehabilitation clinic where the data were assessed.

## Abbreviations

AcAc: Acetoacetate
β-OHB: Beta-hydroxybutyrate
B-Lac: Blood lactate
CER-VD: Commission d’éthique et recherche du canton de Vaud
CHO: Carbohydrate
CRR: Clinique romande de réadaptation
D_max_: Methods for calculating lactate threshold by Bishop et al. [45]
Dmod: Modified D_max_ threshold
FiO2: Fraction of inspired oxygen
UD: Self usual mixed Western-style diet
HR: Heart rate
KD: Ketogenic diet
ICKD: Isocaloric ketogenic diet
KB: Ketobodies
LT: Lactate threshold
P: Power
PaCO_2_: Partial arterial carbodioxide pressure
PaO_2_: Partial arterial oxygen pressure
PiO_2_: Inspiratory oxygen pressure
Q-AAP: Physical activity aptitude questionnaire
RPE: Rating of perceived exertion
SEMS: Sport and Exercise Medicine Switzerland
VO_2_max: Maximal oxygen uptake
